# Assessment of Poorly Compactable Sands by Recycling and Recompaction: Experimental Program and Packing Particle Analysis

**DOI:** 10.3390/ma15238697

**Published:** 2022-12-06

**Authors:** Juana Arias-Trujillo, Agustín Matías-Sánchez

**Affiliations:** Department of Construction, School of Engineering, Universidad de Extremadura, Avda. de la Universidad, s/n, 10003 Caceres, Spain

**Keywords:** poorly compactable sand, recycled, particle packing analysis, void ratios, coordination number, breakage particle index

## Abstract

Compaction is a common ground improvement technique based on the densification of soils for an energy level and optimum water content, mainly influenced by the particle size and curve gradation. Poorly compactable sands, characterized as cohesionless, fine and uniformly graded, are a challenge for earthworks since compaction is not effective due to the lack of a larger range of particle sizes to infill the voids and the compaction energy is not relevant either. These characteristics are common to other materials, i.e., desert sand, industrial or mining by-products or quarry fines, which are mostly discarded to landfill and replaced by proper soils, causing serious environmental issues. To enlarge the technical feasibilities of poorly compactable sands, reducing construction waste and raw material consumption, a mechanical stabilization, based on a repetitive series of recycling and recompaction without binder, is experimentally explored. The behavior observed is also analyzed from reported correlations and a packing particle approach, attending to densification stage, saturation degree, recompaction series, coordination number and packing density. The improvement achieved is moderate and dependent on the cycles applied, showing a characteristic repetitive pattern in the compaction curve, and approaching the estimated minimum void ratio and the theoretical maximum packing possibilities without degradation of the material.

## 1. Introduction

Civil engineering infrastructures request large quantities of different types of raw materials, in particular natural aggregates, from concrete applications [1,2] to earthworks [3], increasing environmental degradation. The climate emergency is a current challenge [4], and therefore, a more sustainable trend should be addressed in the construction sector nowadays, which must be supported by proper technological developments and improvements of the resource efficiency, so engineers and contractors can use more ecofriendly materials and alternative technologies with confidence and security.

Granular materials are commonly used for the construction of plenty of civil engineering works, such as embankment, base and sub-base in highways or soil infilling and leveling. Normally, a soil improvement technique is required to guarantee appropriate geotechnical properties. Depending on each type of soil and the particular characteristic of each infrastructure (loading, permissible settlements, surface and depth of the area to treat, groundwater and water table location), different techniques can be necessary, mainly to control the strength and bearing capacity and compressibility, as well as even to control groundwater issues in the corresponding earth structures [5,6].

Different types of soils can be inappropriate from a geotechnical engineering point of view, such as poorly graduated granular materials, soils with large proportion of fine content, some kinds of clays and silts and organic or contaminated soils, among others, since their mechanical response (strength and deformability) or hydraulic behavior do not satisfy the technical regulations. They are a challenge for construction applications and also an important environmental issue because they are usually discarded to landfill and replaced by other proper soils. However, for a more sustainable approach, this type of materials should be researched for being feasibly considered, with the aim of reducing the volume of construction waste and the raw material consumption.

Earth structures made of fine granular soils are a challenge in geotechnical engineering, in particular with noncohesive soils and uniform particle size distribution. In the literature, many studies established different correlations between compaction parameters and Atterberg limits for cohesive soils [7]. However, the influence of compaction parameters for cohesionless fine granular soils has scarcely been investigated in the literature. As exposed above, some types of soils match to these singular characteristics, such as desert sands, which are widespread in some regions of the world, mainly Africa, Asia and Oceania [8]; or quarry fines, which are obtained as by-products from industrial stone-cutting facilities or mining waste [9,10]. Due to the lack of feasible possibilities, traditional improvement ground techniques have been applied, such as cement stabilization [10,11,12]. For the sake of a more sustainable construction approach, in this research the admixture of any chemical stabilizer or binder has been avoided in order to analyze and enlarge all possibilities of mechanical stabilization in this type of soil.

The most common and widely employed technique is compaction, which is chosen at first option thanks to its simplicity. Compaction consists of densification of the soil by mechanical means, where the soil density is increased by energy applied on the soil, partially removing the intergranular voids. The effectiveness of this technique is controlled by the dry density, since the higher the density, the better the behavior of the soil. The main parameters that affect compaction are the moisture content, the type of soil, the mineralogy, the gradation (grain size distribution) and the energy expenditure [13,14]. The usual compaction tests are the standard Proctor [15] or the modified Proctor [16]; both consist of a very similar procedure, but varying the energy applied: 583 kNm/m^3^ and 2632 kNm/m^3^ (4.5 times higher), respectively. From any compaction test, the optimum water content and the maximum dry density are determined, since for the optimum water content and a particular compaction energy level, the soil achieves the maximum densification stage, and therefore the highest bearing capacity [7]; moreover, its shear strength increases and its compressibility reduces.

Compaction is less effective in uniformly graded noncohesive soils (poorly compactable soil), since the voids between particles are hardly filled by smaller particles due to the lack of them; therefore, the dry bulk density is almost null improved. It can be expected that the compaction properties improve as the compaction energy level is increased, because higher values of maximum dry density occur for modified Proctor than standard Proctor (around 30%) and lower optimum water content (around 40%) [7]. However, these findings are strongly influenced by the type of soils, i.e., Khalid and ur Rehman (2018) [17] reported different experimental correlations between modified and standard Proctor for fine-grained soils depending on the plasticity index. On the other hand, Sulewska and Tymosiak [13] analyzed the compaction response of even-graded medium sand (coefficient of uniformity C_u_ = 3.10, coefficient of curvature C_c_ = 0.99) for both standard and modified Proctor, concluding that the maximum dry densities are close to each other. A similar pattern is also observed for the optimum water content.

The goal of the compaction is the densification of the soil by means of a reduction in void ratio in the structure of the soil. The porosity depends on the particle packing; in particular, for not-aggregated soils, the porosity is entirely dependent on the packing of primary particles [18]. According to Panayiotopoulos [18], the packing behavior can be measured by the minimum (stage of dense random packing) and maximum (stage of loose random packing) void ratios. Different parameters concern the packing of granular materials, such as particle size and particle size range, roundness, sphericity and surface roughness. They can influence void ratio and pore size distribution, the number of points of contact, compressibility, angle of friction and strength of sands, highlighting that particle sizes greater than 0.05 mm have influence on the packing of sands, but not lower size. The moisture content does not affect compaction for narrowly graded sands, except near air-dryness and saturation, where compaction increases.

This research aimed to analyze the response of poorly compactable sands that are mainly characterized as cohesionless, fine particle sizes and uniformly graded material. These characteristics are also presented in other similar materials, which are normally discarded in earthworks, i.e., desert sand, industrial or mining by-products or quarry fines. To reach this goal, the improvement on the compaction properties of cohesionless fine and uniformly particle distributed sand by mechanical stabilization (avoiding any binder) was experimentally tested and analyzed. This mechanical stabilization was developed by a repetitive series of recycling and recompaction of the same material, trying to consider the reiterative application of stress during the construction site lying or traffic load. This characterization was developed experimentally for pre-sieved sand to match to these particular properties. The necessary laboratory tests were conducted to determine the resulting physical and engineering properties. Based on published research [13], the influence of the energy of compaction was not taken into account since it is not relevant for this particular type of soil, and only standard Proctor [15] was considered. In order to assure the durability of this treatment, special attention was paid to the degradation of the material after repetitive cycles of stress, and the possible generation of lower-size particles was also investigated. This improvement was also discussed attending to the packing particles of the soil by different models, and the corresponding relationships with the variation of the maximum and minimum void ratios. Finally, the main conclusions are addressed. 

## 2. Materials and Methods

### 2.1. Material

The material tested in this research corresponds to commercial river sand, commonly used for concrete or pavements, supplied in Caceres (Spain). This is collected, screened and washed, where all clay and silts contents are removed before supplying. This material is no contaminated by organic matter, heavy metal or any other industrial by-product or demolition waste. Therefore, this sand is a cohesionless material, and from a mineralogical point of view, the composition is mostly formed by silica.

### 2.2. Composition of the Particle Size Distribution

**Figure 1:**
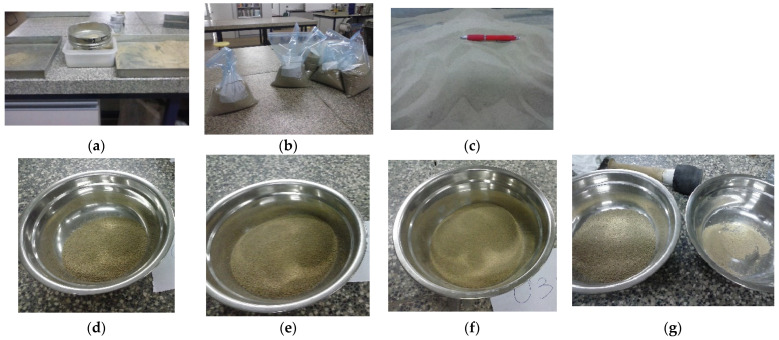
Preparation of the tested sand sample: (**a**) particles larger than 1.25 mm sieve removed; (**b**) bags classified with each particle size fraction; (**c**) final sample of sand tested after mixing the proper amount of the corresponding sieves; (**d**) 0.63 mm sieve; (**e**) 0.4 mm sieve; (**f**) 0.32 mm sieve; (**g**) 0.16 mm and 0.08 mm sieves.

This research is focused on fine and not well-graded soils; therefore, the particle size distribution was properly selected by sieving analysis [19], mainly ranging between 0.08 and 0.63 mm. To guarantee these properties, the whole amount of sand was uniformly mixed and quartered to divide and obtain a representative sample fraction. After that, the sample was sieved and the particles larger than 1.25 mm sieve were removed (Figure 1). Then, small amounts of material were successively sieving, separating the corresponding masses of each sieve, as shown in Figure 1. The final particle size distribution was composed by adding the appropriate mass of each sieve in order to obtain the typical particle size distribution of poorly compactable sand. The gradation obtained is similar to the particle size distribution of other cohesionless fine uniform sands such as desert sand [8,11]. 

The cumulative %-weight passing after sieving analysis of the tested material is presented in Table 1. The maximum particle size is 0.63 mm and the fine content is 1.81%. Due to the scarce amount of fine fraction, only dry sieving analysis was considered [19]. According to the USCS classification system [20], this sand can be classified as poorly graded (SP), and according to AASHTO system [21] as A3. The coefficient of uniformity is C_u_ = 2.03 and the coefficient of curvature is C_c_ = 0.82. The main physical characteristics of this material are listed in Table 2.

### 2.3. Experimental Procedure

The experimental testing program is defined to analyze the improvement on the compaction properties by means of the mechanical stabilization proposed, which is based on repetitive series of recycling and recompaction of the material samples without chemical binders. Moreover, the influence of several cycles of recompaction energy in the degradation of sand, and the influence of this possible degradation in the compaction response, are also checked. The experimental works were carried out in the Geotechnical Laboratory at the University of Extremadura (Caceres, Spain).

The experimental properties investigated are moisture content–dry density relationship and sieving size particle. The compaction test followed is the standard Proctor [15], and the sieving analyzes were developed according to [19]. Both experimental procedures have been successfully validated in many civil engineering works.

#### 2.3.1. Compaction Test

The compaction test is aimed at obtaining the relationship between the maximum dry density of soil and the optimal water content, for a specific energy of compaction, whereby densification occurs in the soil, reducing the void content. The maximum dry density is related to the behavior of infills, embankments or layers of pavements, since higher dry density, higher strength and lower susceptibility to volume changes (lower deformability) are expected.

For the compaction procedure, the standard Proctor [15] was adopted. This procedure requires significantly less material than modified Proctor [16], whereas similar results are obtained in the case of even-graded medium sand between both procedures [13]. Moreover, the maxim particle size allowed for this test is 20 mm, while the maximum particle in the tested sand is 0.63 mm. The dimensions of the tested specimens and the compaction energy characteristics are included in Table 3, where the energy applied by unitary volume is 583 kNm/m^3^ or 0.583 J/cm^3^.

**Figure 2:**
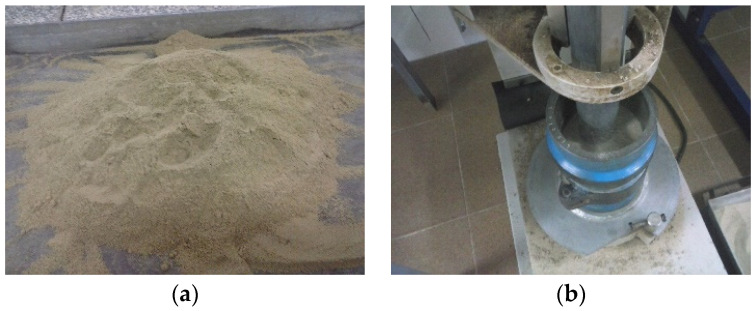

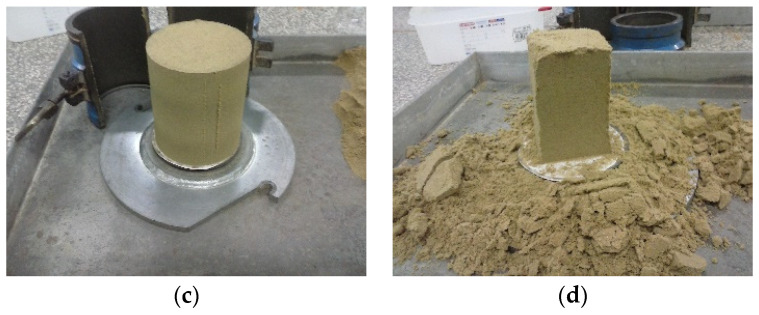
Proctor compaction procedure: (**a**) uniform mixing of the sample for the corresponding water content; (**b**) compacted specimen in an automatic compactor; (**c**) demolding of the compacted specimen; (**d**) cutting the specimen to obtain a representative sample to obtain the moisture content.

For all the specimens tested, the compaction was applied by means of an automatic compactor. Compaction curves were developed, ranging the water content between 0% and 17%, approximately, with tentative increments between 1% to 4% in order to characterize both the very low values of dry density, corresponding to scarce water content, and the highest dry density, corresponding to the optimum water content [8]. The procedure is summarized in Figure 2.

#### 2.3.2. Recycling and Recompaction of the Material

The mechanical stabilization of the material was carried out by means of different successive series of recycling and recompaction of the same material. A total of four series of standard Proctor energy tests were developed. Each compaction series was restarted after 5 or 6 compaction curve points and the specimens were kneaded and tested in increasing order of water content, assuming as initial water content the value estimated in the previous specimen (Table 4). The exact value of water content was exactly measured from a small sample obtained from the core of each specimen, which was dried in oven, according to [22]. The exact values of water content are used hereafter. For comparison purposes, before the starting of a new series of proctor curves, the whole amount of material was completely dried in oven to assure a null moisture content, and it was crumbled to prevent caking, manually or with a rubber mallet.

#### 2.3.3. Re-Sieving Analysis

After the fourth series of compaction, the whole amount of material was sieved newly. This postsieving analysis allows us to determine how the different series of recycling and recompaction alter the particle size distribution and the possible degradation of the material.

## 3. Results and Discussion

### 3.1. Compaction Curve: Dry Density and Water Content Relationship

**Figure 3:**
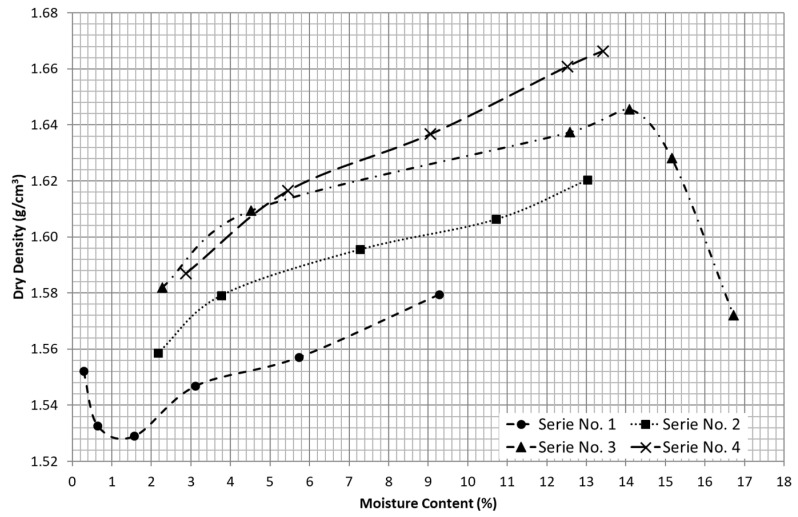
Compaction curves for each series tested.

The relationship obtained for each specimen tested between dry density and %-moisture content is shown in Figure 3. In series No. 1, a dry density (1.55 g/cm^3^) associated with a very low value of moisture content (0.28%) can also be observed herein. This behavior can also be observed in some types of fine soils with uniform particle size distribution [8]. From the range of water content established for this series, it was not possible to achieve the wet side of the compaction curve and therefore the maximum dry density.

Series No. 2 and 3 were developed for a similar range of water content in the dry side of the compaction curves. Both series present a quasi-parallel and equidistant trend to respect the first series. In the case of the third series, the maximum dry density was achieved for 1.646 g/cm^3^ and 14.09% for optimum moisture content. Moreover, it can be observed that the slope of the dry side of the compaction curve is less steep than the wet side, so this material can be highly influenced by the excess of moisture content after reaching the optimum.

Finally, series No. 4 presents an almost coincident response to the series No. 3 for the very beginning of the compaction curve (low values of moisture content). However, as the moisture content is increased, the compaction curve tends to be equidistant to the previous ones, increasing the values of dry density.

After several cycles of recycling and recompaction of the material, a quasi-equidistant translation of the dry side of the compaction curve towards higher values of dry densities can be observed, ranging from 0.024 to 0.031 g/cm^3^ by series, which represents a relative increment in dry density of around 1% and 2%. Although the increments in dry density are moderate, the maximum dry density that can be achieved seems to be improved after this mechanical stabilization. On the other hand, the range of water content— and in particular, the optimum moisture content—seem to not be significantly altered by the number of recycling and recompaction series.

The range of values of maximum dry density and optimum moisture content obtained for this material are in agreement with the experimental data reported by other studies on sandy materials with similar particle size distributions and C_u_ and C_c_ values, carried out both with standard Proctor [13,23] and with modified Proctor [11,13]. However, these values are slightly lower than the maximum dry density estimated from most of the correlations reported by [7], which is around 1.82 g/cm^3^ for soils from clays to sandy soils with gravels lower than 4.76 mm, for coal ashes and soil and for fine-grained soils. Therefore, it can be concluded that for cohesionless, fine and uniformly graded sands, the maximum dry density obtained experimentally is lower than the theoretical values excepted, and higher compaction energy levels do not significantly improve the compaction results for this material.

### 3.2. Degree of Saturation

The theoretical maximum value of dry density of a soil is reached when all the air has been removed from the void spaces. This occurs for the complete saturation of the soil, i.e., when the degree of saturation is equal to 100%, defined by the zero air void line. This stage cannot be achieved by the compaction procedure, and therefore, the compaction curves are always below the zero air void curve.

**Figure 4:**
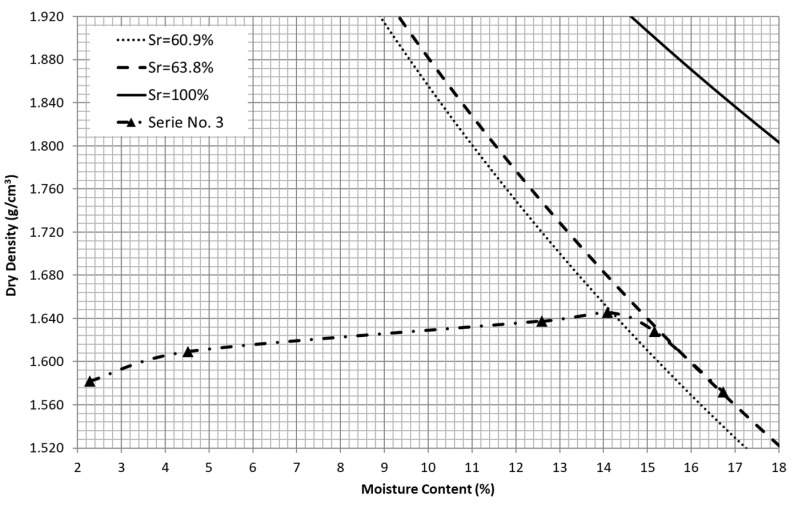
Compaction curve corresponding to series No. 3 and degree of saturation lines for optimum value (S_r_ = 60.9%), for wet side (S_r_ = 63.8%) and zero air void (S_r_ = 100%).

In Figure 4, different degrees of saturation of the tested sand are depicted from the compaction curve of series No. 3, from which the wet side of the compaction curve was also obtained. The degree of saturation for the optimum water content–maximum dry density is equal to 60.9%, very close to the value of 60.4% obtained from the reported correlation between the optimum degree of saturation and the optimum water content published by Spagnoli and Shimobe [7]. Moreover, from the experimental results it can be observed that the compaction points corresponding to the wet side match with those corresponding to a degree of saturation about 63.8%. For comparison purposes, the zero air void curve (A_v_ = 0) or degree of saturation equal to 100% (S_r_ = 100%) was also drawn.

This reveals that this type of material presents a threshold for the degree of saturation, which corresponds to the wet compaction side that cannot be exceeded despite the water content. Moreover, it can be observed that the degree of saturation reached for the optimum compaction stage is very close to this threshold. Therefore, as it has been highlighted above, the compaction properties of this material are very sensitive to the excess of water. In general, the degree of saturation for the optimum compaction stage of soils is around 85% [7], which is significantly higher than 60.4%, obtained for the tested sand. Moreover, Spagnoli and Shimobe [7] reported that for soils with an optimum water content lower than 40%, the soil type or the particle size can remarkably affect the relation between the degree of saturation and the optimum water content. These support the claim that cohesionless, fine and uniformly graded sands are poorly compactable materials.

### 3.3. Changes in Particle Size Distribution after Recycling and Recompaction Series

**Figure 5:**
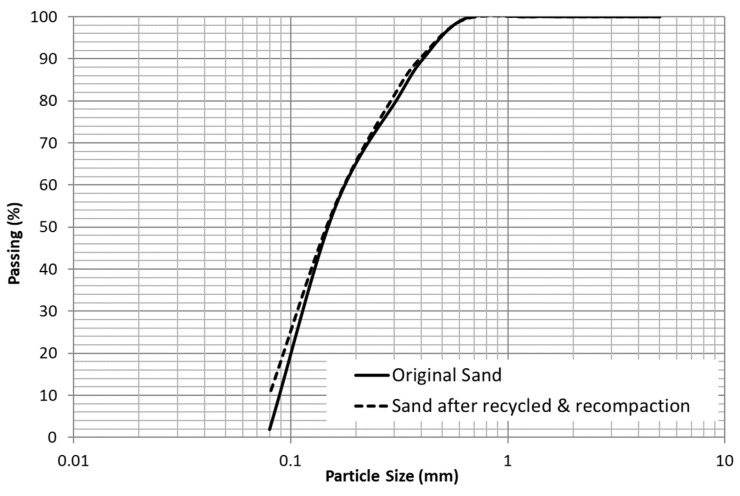
Particle size distributions of the original sand and the sand after four series of recycling and recompaction.

**Figure 6:**
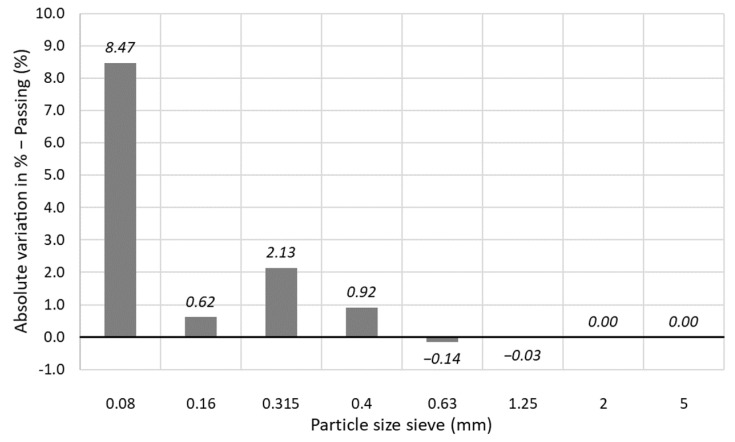
Histogram of the absolute variations for each particle size of the altered sand (after testing) in respect to the original material.

The alteration in the particle size distributions of the material after the four repetitive series of recycling and recompaction of the material are compared in Figure 5. A not significant variation between both curves can be observed, highlighting the most relevant differences in the smaller sieves. The absolute variations for each particle size fraction, computed as the subtraction between the altered sand with respect to the original sand, are plotted in the histogram in Figure 6. In this figure, very slight reductions can be observed for the bigger sieves (mainly #0.63 mm), whereas an increment is observed in the rest of fractions. The highest increment occurs for particle sizes lower than 0.08 mm, increasing by almost 8.5%. The variation of the gradation properties of the sand, such as C_u_, C_c_ and D-values, are summarized in Table 5, where a slight increment in C_u_ can be observed, whereas the rest of the parameters do not vary significantly. This reveals that the degradation of the particles due to the successive series of recycling and recompaction of the material is reduced, resulting into a moderate increment in finer particles, especially of the smallest size, whereas the largest sizes scarcely vary, involving a slight alteration in the gradation properties of the sand.

### 3.4. Breakage Particle Index

The possible degradation of the material can be of relevant interest in practical construction applications. The compaction procedure can induce crushing and particle breakage; therefore, the well-known method proposed by Hardin [24] was adopted, where the degradation of a material after a stress stage can be quantified by comparing the evolution of the particle size distributions. The breakage particle index, Br, proposed by Hardin, considers the area delimited between the initial and the current gradations in respect to the area under the initial particle size distribution. The higher the Br, the more that degradation of the soil occurs after testing. Hardin assumed the crushing of a soil up to particles of 0.074 mm, since the very small particles are less susceptible to be broken. In this research, Hardin’s threshold was established up to 0.08 mm. For the material analyzed in this research, the breakage particle index is nearly null, approximately 0.15%.

### 3.5. Estimation of Limit Index Void Ratios

The packing behavior of sands can be measured by the minimum and maximum void ratios, which correspond to a stage of dense random packing and a stage of loose random packing, respectively [18]. Due to the complexity of testing cohesionless samples, in both the loosest and densest stages at lab, and the significant variabilities that can be obtained in the experimental data [25,26], the limit index void ratios were estimated by means of several correlations reported in the literature for nonplastic fine-content sands, which are computed in Table 6. Many studies have established empirical or probabilistic correlations among some index properties, i.e., particle size (i.e., D_50_), particle shape, fine content (FC), roundness, coefficient of uniformity (C_u_), etc., from experimental tests or collected from the literature by means of single-variable or multivariable functions. A large review can be found, for example, in [27,28,29].

The limit index void ratios included in Table 6 were estimated for comparative purposes. Most of the models were considered as input parameters D_50_ and/or FC. The range of values obtained from these models fits properly between all of them. For the cohesionless, fine and uniformly graded sand tested in this research, the range of maximum void ratio (e_max_) varies approximately 0.71 to 1.29, and the minimum void ratio (e_min_) varies between 0.48 to 0.84.

For the following particle packing analysis included in this research, the void ratios considered were estimated by the third-degree polynomial equation fitted by Yilmaz [27], after testing a large range of artificial graded sands, because the particle size distribution can be best matched. The material employed by Yilmaz corresponds to a commercial silica sand with a specific gravity equal to 2.68, with a subangular shape. From the large number of series tested by Yilmaz, defined according to the Unified Soil Classification System (USCS), series No. 80–No. 200, without mixing with any other series of graded material (C_u_ = 1.375 and C_c_ = 0.92), is the best fitted to the material analyzed in this research. Therefore, the maximum and minimum void ratios estimated are e_max_ = 1.085 and e_min_ = 0.64, and the range of void ratio (e_max_–e_min_) is 0.445 computed from the Yilmaz’s correlations. The results obtained agree with the range of values presented in Table 6. Applying the index property expressions shown in Equations (1) and (2), the porosity (n), the dry density (γ_d_) and the packing density (D) can be calculated from the void ratio (e) and the density of solids particles (γ_s_), which are listed in Table 7.

(1) $$e=\frac{n}{1-n}$$

(2) $$n=\frac{e}{1+e}=1-\frac{\gamma_{d}}{\gamma_{s}}=1-D$$

Along the different compaction series developed, the lowest γ_d_ obtained is 1.53 g/cm^3^, which implies that the corresponding void ratio is e = 0.75 (n = 0.43; D = 0.57), computed from Equations (1) and (2). This is the loosest stage of the sand reached experimentally, although the tested result obtained does not fit to Yilmaz’s values (Table 7), revealing that this theoretical loosest stage cannot be achieved in the practice for this sand.

On the other hand, from the experimental results, it can be observed that as the moisture content is approached the optimum, and for the successive recycling and recompaction series, the dry density gradually increased up to 1.66 g/cm^3^ (e = 0.61; n = 0.38; D = 0.62 computed from Equations (1) and (2)). These results perfectly approach the maximum dry density corresponding to the minimum void ratio estimated from Yilmaz (γ_d_ = 1.63 g/cm^3^ and e_min_ = 0.64). Therefore, the mechanical stabilization based on successive recycling and recompaction series of the sand promotes the possibility of achieving the theoretical dense random packing (densest stage) estimated from Yilmaz’s regressions.

### 3.6. Particle Packing Analysis

In many geotechnical applications, the effectiveness of compaction is measured by means of the increment in dry density. The compaction technique tries to reduce the pore space in the soil sample varying the moisture content, and therefore increasing the packing of the solid particles. Therefore, particle packing of soils affects their physical and engineering properties. The improvement achieved after the mechanical stabilization procedure described previously was also characterized by a particle packing model and the limit index void ratios.

For not-aggregated soils (single-grain) such as sandy and coarse silty soils, the porosity is entirely dependent on the packing of the primary particles [18]. For sands, the packing particles and the actual values of minimum and maximum void ratio index can be influenced by the particle size and the particle size distribution, the fine contents, the sphericity, the roundness and the surface roughness of solid particles [18,26]. In this research, special attention is paid to the most relevant topics such as particle size, fine content and particle size range.

The soil investigated herein is a cohesionless uniformly graded sand where the particle size range is very narrow and most of the grains present a small particle size, resulting in a nonplastic fine sand. In the packing arrangements of the fine particles of a soil, the forces of attraction–repulsion can have more influence than gravity forces for these types of particles, since they try to adhere to one another and hinder the slipping of the fine particles into the voids, resulting in looser structures with higher void ratios [26], enhancing in water conditions, and therefore, loose random packing may not be applicable [18]. This phenomenon is relevant for particles below 0.05 mm or 0.08 mm [18,26]. In this research, since the fine content is reduced, the tested sand is at the limit of these thresholds, so the interparticle forces are neglected.

#### 3.6.1. Single-Size Particle Packing

For the packing analysis, the particles of the sand investigated can be assumed as spherical particles because the variety of particle sizes is scarce. In random packing of sphere particles, the coordination number (N) is a relevant parameter that defines the number of particles in contact with any given particle. Although the pattern of random packing is usually unknown in advance, in the literature [18,26], five possible regular geometric arrangements are usually considered. The five main types of packing for spherical particles with the same diameter are: simple cubic, cubical-tetrahedral or single stagger, tetragonal-sphenoidal or double stagger, pyramidal and tetrahedral. The coordination number and the packing density increase from 6 and 0.5236 (simple cubic) to 12 and 0.7405 (pyramidal or tetrahedral), respectively, whereas the void ratio reduces from 0.9099 (simple cubic) to 0.3504 (pyramidal or tetrahedral). Attending the Kepler conjecture, the maximum packing density of equally sized spherical particles is not greater than π/√18 (around 0.74048) and a coordination number equal to 12, which corresponds to the two last types of packing listed.

From the experimental test of packing uniform spheres [32] with diameters ranging from 0.041 mm to 0.493 mm, the void ratio ranges between 0.60 (D = 0.625; N = 9.32) to 0.68 (D = 0.59; N = 8.46), independently of the diameter or the particle density. Moreover, the experimental test of randomly packing steel balls of diameter equal to 3.17 mm showed that the void ratio varied between two limit stages, named as loose and dense random packing with void ratios varying 0.66 to 0.57, respectively [33]. The packing density varies from 0.601 to 0.637, and the coordination number from 8.6 to 9.6, respectively [18]. Therefore, two packing limits can be assumed, which correspond to dense and loose random packing, relating with the minimum and maximum void ratios, respectively [18]. For a random packing of spheres, the porosity can be related with the coordination number by the Equation (3) [18,34].

(3) $$N=26.486-\frac{10.726}{1-n}$$

**Figure 7:**
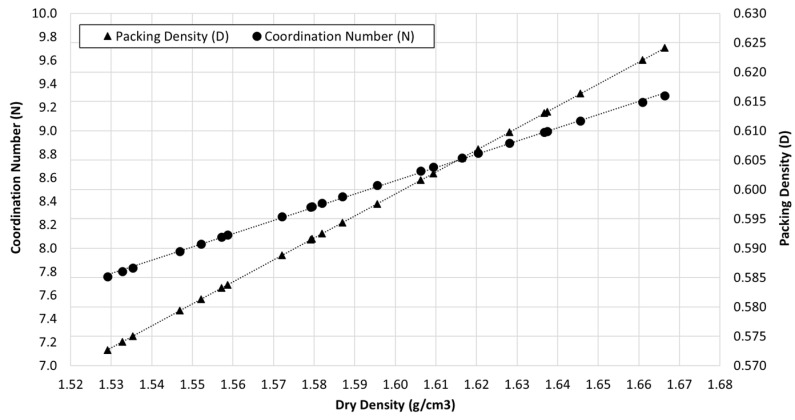
Variation of coordination number (N) for the values of dry density obtained after the compaction tests and adjusted trend expression.

The coordination number (N) and the packing density (D) were calculated from the compaction results obtained, as shown in Figure 7. From this figure, a positive linear relationship can be outlined between coordination number (N) and dry density and between packing density (D) and dry density, since as the density increases, N and D range from N = 7.57 and D = 0.574 for the lowest dry density to N = 9.3 and D = 0.624 for the highest dry density tested, which represent an increment equal to 22% and 8.7%, respectively. This finding is consistent with the traditional approach of the compaction technique since the arrangement of the particles improves with the increment in density, resulting in more points of contact between particles. However, the highest coordination number obtained is still far from 12, which corresponds to the maximum packing density of an idealized distribution of uniform spherical particles, but very close to the dense random packing reported experimentally [18,32,33] and to the minimum void ratio estimated from Yilmaz (e_min_ = 0.64; N = 8.9; D = 0.61) [27].

**Figure 8:**
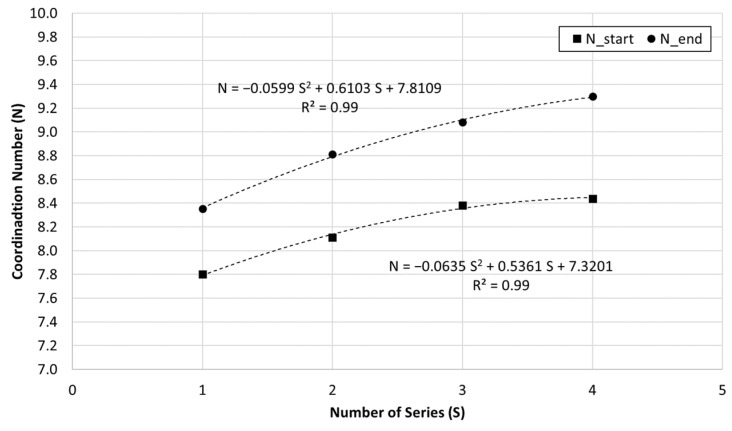
Variation of the coordination number (N) at the start and at the end of each series and the corresponding adjusted trend expressions.

The evolution of the coordination number is also analyzed for each series of recycling and recompaction, regarding the dry density obtained for the first and last points of each recycling and recompaction series in the dry side of the compaction curve, Figure 8. It must be highlighted that the water content for the first point of each series is similar, between 2% and 3% at Series No. 2, 3 and 4, and below 1% at Series No. 1, and the energy of compaction after each series is also similar. From Figure 8, it can be observed that coordination number estimated for the first point of each series (N_start) is higher as the successive series of recycling and recompaction occurred, despite the initial water content being similar for all of them. The coordination number corresponding to the final point of each series shows a similar tendency. The increment in the coordination number of each series in respect to each initial value of N ranges between 7% to 10%, independent of the water content or dry density achieved in the experimental tests.

#### 3.6.2. Various-Size Particle Packing

Mixtures of different-size particles have also been studied theoretically and experimentally in the literature for many topics of interest [18,26,35,36,37]. In general, it is assumed that the particles are spheres and the minimum void ratios are independent of the grain diameter, and the specific gravity does not vary with the particle size [18,26]. Moreover, the variation of the void ratio with the percentage of fine particles (small particles) and the effect of the diameter ratio are two traditional issues of relevant interest [26].

For a mixture of two spherical-sized particles, when the finer content is increased, the voids can be filled, reducing the void ratio and increasing the dry density. The minimum void ratio is reached when the voids between coarse fraction are completely full of small particles, which can be achieved for an optimum fine content. If the fine content increases beyond this value, the large particles are spread apart from each other, increasing the void ratio [18,26,32,37]. The optimum fine content, which corresponds to the minimum void ratio, is a theoretical value that cannot be achieved in practice since the diameter of the fine particle should be infinitely small [26].

For the influence of the fine content described above, it is assumed that the fine particles are small enough, presenting a large ratio between the large diameters and the small diameters. If the ratio between particles is not enough, the small grains cannot fill the voids between large grains [32]. The most effective reduction in the minimum void ratio was observed for diameter ratios below than 7; for upper values, the reduction is still maintained but it is not so effective [26]. In the experimental tests developed by Lade et al. [26] with Nevada sand (fine-grained quartz sand with nonplastic fines) to study the range of limit index void ratios for various-size particle mixtures, three intervals of sizes were considered: ASTM No 50-No 80 (0.3 to 0.18 mm); ASTM No 80-No 200 (0.18 to 0.075 mm) and fine particles (smaller than 0.075 mm). For the combination of any of the two larger sizes with the fine content, they revealed that the minimum and maximum void ratios follow similar trends, although lower values of void ratios were obtained for the combination of the largest fraction with the fine content. As the diameter ratio is lower, its influence on the minimum void ratio is less evident, and the fine content is less relevant [26].

More recent packing particle models reported in the literature try to consider additional particle interactions to the previous ones (filling and occupying effects) that affect the manner that particles of different sizes are packed, especially for binary mixture with varying size ratios. These particle interactions are [35,36]: the loosening effect (the fine particles squeeze themselves into the voids of the coarse particles); the wall effect (the coarse particles disrupt the packing of the fine particles at the wall-like boundaries of the coarse particles); and the wedging effect (when the fine particles are slightly less than enough to fill the voids, some isolated fine particles could be entrapped in the gaps between the coarse particles, wedging them apart, or when the fine particles are slightly more, some gaps between the coarse particles could be incompletely filled by fine particles, causing voids there, and the apparent wedging of the fine particles against the coarse particles). These three particle interactions decrease the packing density. Empirical models defined by two or three parameters can be found in the literature trying to consider all these effects independently [35,36].

Since the material tested in this research presents a very uniform particle size distribution, it is analyzed as a binary mixture of spherical particles (fine and large fractions) according to the two-parameter (Model-2p) and three-parameter (Model-3p) models reported by Chan et al. [35,36]. Besides the filling and occupying effects, the Model-2p considers the loosening and wall effects, and the Model-3p also considers the wedging effect. The optimum values of each fraction sizes that yield the maximum packing density can also be obtained from both models. The three parameters are experimentally fitted and they depend on the size ratio s, defined as the ratio of the size of the fine particles in respect to the size of the large particles. After analyzing the experimental results of a wide range of size ratios from 0.02 to 0.74, the authors concluded that both models are sufficiently accurate when the size ratio is larger than 0.65. For lower values, the two-parameter models would overestimate the packing density, mainly as close to the optimum fractions to reach the maximum packing density, whereas the three-parameter model was the most accurate.

In regard to the particle size distribution of the sand tested in this research, the fine particle size was established as 0.08 mm, since more than the half of the sample was retained by this sieve, whereas the large particle size was estimated as the weighted arithmetic mean from particles ranging from 0.63 mm to 0.16 mm sieves, resulting a large fraction equal to 0.247 mm, and therefore, a size ratio s = 0.324. For this research, the same type of packing and the same solid density were assumed for both sizes of grains.

**Figure 9:**
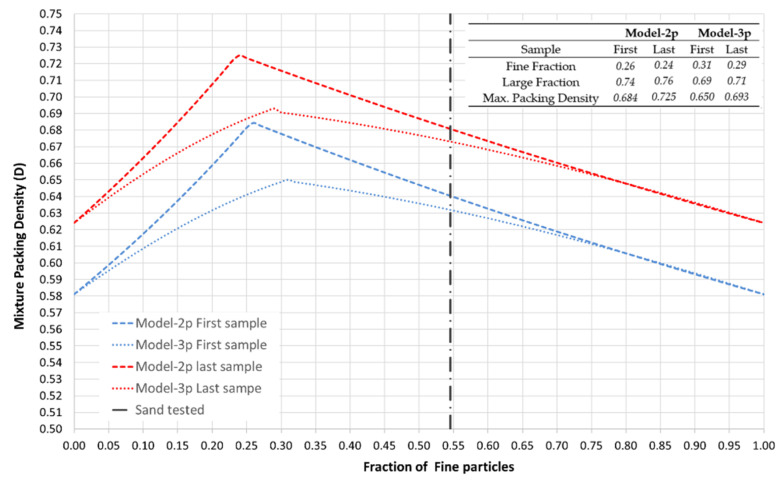
Variation of the mixture packing density with the fraction of fine particles estimated for Model-2p and the Model-3p [35,36] assuming the type of packing corresponding to the first (first point of Series No. 1) and the last (last point of Series No. 4) samples tested. The theoretical maximum packing density and the corresponding optimum fine and large fractions are also tabulated.

**Figure 10:**
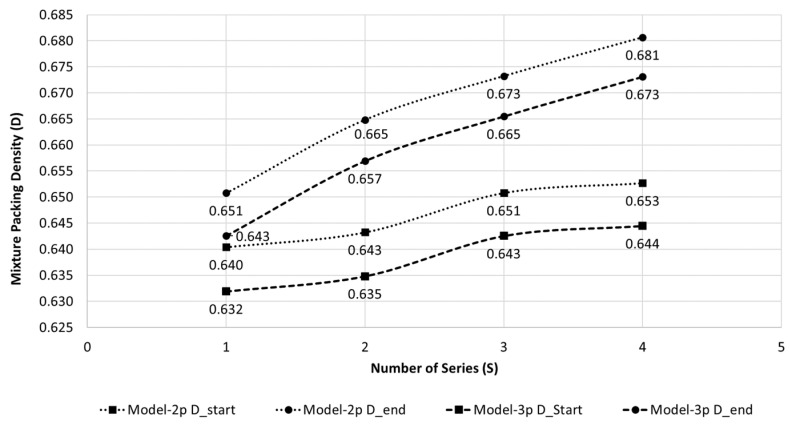
Variation of the mixture packing density (D) for each recycling and recompaction series computed for Model-2p and Model-3p [35,36].

Figure 9 shows the variation of the mixture packing density with the fraction of the fine particles ranging from 0 to 1 (the corresponding large fraction can be calculated as the difference up to one) computed by Model-2p and Model-3p according to the expressions reported by [35,36] for the characteristics of the tested sand. The type of packing corresponding to the first (first point of Series No. 1) and the last (last point of Series No. 4) compaction samples was considered, since they represent the extreme values experimentally tested. These curves also lay out the theoretical maximum packing densities and the corresponding optimum fractions of each size, whose values are tabulated in the figure. The fine fraction of the tested sand is highlighted by a dotted vertical line as reference. On the other hand, the evolution of the mixture packing density for the packing obtained at the beginning and ending of each recycling and recompaction series is plotted in Figure 10 for both models.

From Figure 9 and Figure 10, it can be observed that Model-2p shows mixture packing densities slightly higher than Model-3p. Despite the size ratio of the sand being lower than 0.65, the results obtained for Model-2p can be reliable because the fraction values are far from the optimal ones. The optimum fractions of each size of the tested sand are around 25% of fine and 75% of large fractions for Model-2p, and 30% and 70%, respectively, for Model-3p, to achieve the theoretical maximum packing density, which ranges between 0.650 to 0.725. The theoretical maximum packing density of the original stage of sand is around 0.65 (Model-3p). This result agrees with the denser packing density corresponding to the minimum void ratio estimated from Yilmaz for this sand [27]. The real fraction composition of the tested sand is far from these optimum values, which asserts the poor compaction properties of this material. However, it can be observed that thanks to the mechanical stabilization employed in this research, the mixture packing density is increased, similarly to the pattern observed with the coordination number (Figure 8). In Figure 9, it can be observed that the packing densities at the beginning of each series are higher than the previous series, achieving an increment of around 2% after the four series. This increment is more relevant if the ends of each series are compared, showing an increment of around 4.5% in the mixture packing density after the four series. The mixture packing density experimentally obtained after the four series is very close to—or even slightly higher than—the theoretical maximum packing density expected for the original stage of the sand.

## 4. Conclusions

The improvement on the compaction properties of a fine uniform and cohesionless sand, which presents strong similarities with other materials, i.e., desert sand or industrial or mining by-products, were tested, and the results were analyzed from a packing particle approach. Compaction, which is the most common densification procedure, is not so effective for this type of materials due to the lack of smaller particles to infill the voids, and they are usually discarded to landfill because of their lousy engineering behavior. For the sake of a more sustainable construction, a mechanical stabilization based on a repetitive series of recycling and recompaction is explored, omitting any type of binder. Following this, the most relevant findings are pointed out.

- After each series of recycling and recompaction of the material, an equidistant translation of the dry side of the compaction curve towards higher values of dry densities can be observed, increasing by around 1–2% by series, whereas the range of moisture content is not significantly altered.
- The degree of saturation on the wet side of the compaction curve (about S_r_ = 63.8%) is very close to the corresponding degree of saturation for the optimum compaction stage of the sand (S_r_ = 60.5%), so the compaction response of this material can be very sensible to excess of water.
- The degradation of the material is very reduced, with Hardin’s breakage particle index almost null. The fractions of particles lower than 0.08 mm are slightly incremented, although the reduction in the bigger sieves is very limited.
- The theoretical dense random packing (densest stage) estimated from Yilmaz’s regressions [27] is achieved experimentally (γ_d_ = 1.66 g/cm^3^), whereas the loosest stage is not, obtaining a higher densification stage (γ_d_ = 1.53 g/cm^3^).
- Assuming a packing of uniform spheres, a positive linear relationship can be outlined between the coordination number (N) and the packing density (D) in respect to dry density. N is increased between 7% to 10% after each series of recycling and recompaction, independently of the moisture content or dry density.
- The tested sand is also modeled as a binary mixture of spherical particles (55% fine and 45% large fractions; size ratio s = 0.324) by two-parameter and a three-parameter models that take into account the loosening, wall and wedging effects [35,36]. The theoretical optimum fractions (Model-2p: 25% fine, 75% large; Model-3p: 30% fine, 70% large) to achieve the theoretical maximum packing density for this material (up to 0.725) are far from the real composition of the sand, which presents a theoretical maximum packing density equal to 0.65 (Model-3p), asserting the lousy compaction properties of this material. After the mechanical stabilization is researched, the mixture packing density increases by up to 4.5%, matching the theoretical maximum packing density expected for the original sand.

## Figures and Tables

**Table 1:** Cumulative %-weight passing after sieving analysis [19].

Sieve Size (mm)	%-Weight Passing
5	100
2	100
1.25	100
0.63	99.49
0.4	89.52
0.32	81.11
0.16	54.52
0.08	1.81

**Table 2:** Properties of tested sand sample.

Sand Properties	Value
Specific gravity (G_s_)	2.67
Natural moisture content	Null
D_10_: grain diameter at 10% passing (mm)	0.089
D_30_: grain diameter at 30% passing (mm)	0.116
D_50_: grain diameter at 50% passing (mm)	0.150
_D60:_ grain diameter at 60% passing (mm)	0.184
C_u_: coefficient of uniformity	2.06
C_c_: coefficient of curvature	0.82
Color	Light color
Classification soil (USCS) [20]	SP-Poorly graded sand
Classification soil (AASHTO) [21]	A3

**Table 3:** Characteristics of compaction procedure according to standard Proctor [15].

Characteristic of Tested Specimen	Value
Diameter of the specimen (mm)	102
Height of the specimen (mm)	122.4
Volume of material (cm^3^)	1000
Hammer falling elevation (mm)	305
Hammer mass (kg)	2.5
Number of layers	3
Blows by layer	26
Compaction energy (J/cm^3^)	0.583

**Table 4:** Series of compaction tested, number of specimens and range of moisture content.

No. Series of Compaction	Number of Specimens	Range of Water Content Tested
1	6	0.28–9.27%
2	5	2.17–13.03%
3	6	2.28–16.73%
4	5	2.87–13.42%

**Table 5:** Variation of the sand gradation properties after testing.

Gradation Properties	Original Stage	Altered Soil after Testing
D_10_ (mm)	0.089	0.08
D_30_ (mm)	0.116	0.109
D_50_ (mm)	0.150	0.135
D_60_ (mm)	0.184	0.180
C_u_	2.06	2.25
C_c_	0.82	0.82

**Table 6:** Limit index void ratios for the tested sand estimated, as reference, from empirical models reported in the literature.

Reference	Input Parameter	e_max_	e_min_	Remark
Patra et al. [30]	D_50_	1.06	0.84	Uniform and nonuniform river sands, with C_u_ = 1.42 to 9.83
Bradley and Cubrinovski [31]	D_50_, FC	1.29	0.79	Sandy and silty soils with FC ≤ 30%
Chang et al. [29]	D_50_	1.01	0.62	Uniform sands
Cetin and Ilgar [28]				From nonplastic silts to fine to coarse sand and gravels:
	D_50_	0.91–1.23	0.54–0.70	Probabilistic model No. 1
	D_50_, FC	0.71–1.08	0.48–0.65	Probabilistic model No. 2

**Table 7:** Limit index voids for the tested sand estimated from Yilmaz [27].

Limit Void Ratios	n	D	γ_d_ (g/cm^3^)
e_max_ = 1.085	0.52	0.48	1.28 (Loosest stage)
e_min_ = 0.64	0.39	0.61	1.63 (Densest stage)

## Data Availability

Data are contained within the article.
